# Nomenclature and placental mammal phylogeny

**DOI:** 10.1186/1471-2148-10-102

**Published:** 2010-04-20

**Authors:** Robert J Asher, Kristofer M Helgen

**Affiliations:** 1 Museum of Zoology, University of Cambridge, Downing St, CB2 3EJ UK; 2 National Museum of Natural History, Smithsonian Institution, P.O. Box 37012, MRC 108 Washington, DC 20013-7012 USA

## Abstract

An issue arising from recent progress in establishing the placental mammal Tree of Life concerns the nomenclature of high-level clades. Fortunately, there are now several well-supported clades among extant mammals that require unambiguous, stable names. Although the International Code of Zoological Nomenclature does not apply above the Linnean rank of family, and while consensus on the adoption of competing systems of nomenclature does not yet exist, there is a clear, historical basis upon which to arbitrate among competing names for high-level mammalian clades. Here, we recommend application of the principles of priority and stability, as laid down by G.G. Simpson in 1945, to discriminate among proposed names for high-level taxa. We apply these principles to specific cases among placental mammals with broad relevance for taxonomy, and close with particular emphasis on the Afrotherian family Tenrecidae. We conclude that no matter how reconstructions of the Tree of Life change in years to come, systematists should apply new names reluctantly, deferring to those already published and maximizing consistency with existing nomenclature.

## Background

The last decade has witnessed an unprecedented increase in the stability of the mammalian Tree of Life [e.g., [1-4]]. Although not all clades are fully resolved, and our understanding of many extinct radiations remains poor, several previously intractable issues surrounding the living radiations have now been settled [see review in [5]]. A consequence of this newfound stability is the need to establish names for several high-level clades. In current practice, the attribution of scientific names to groups of organisms relies on common descent as the underlying biological principle. When a previously unrecognized pattern of descent is discovered, it deserves to be epitomized by a coherent and legal taxonomic name. Ideally, such a name should be familiar to its users, related etymologically to the group in question, not easily confused with other names, and grammatically correct. Taxonomic convention at or below the Linnean Family is regulated by the International Code of Zoological Nomenclature (or ICZN) [6], which provides a legal recourse for resolving the many ambiguous and controversial cases that occur in zoological nomenclature [7]. This code arbitrates among the competing demands of the scientific community and, ideally, maximizes coherence in animal nomenclature.

At the family level and below, Linnean categories require types (genera for families, species for genera, specimens for species). Hypotheses regarding phylogenetic dispersion around the type form the anchor points from which synonymies can spring. Because the ICZN does not apply to units of the Linnean hierarchy above the family, high-level nomenclature does not have at its core the type concept. This is a fundamental difference between low- and high-level taxonomy. Not only are high-level taxa not associated with type grounding, but they are also freed from requirements of diagnosis/description, a requirement at least in the vaguest sense for establishment/availability of lower-level names. In practice, it is a hypothesis of common ancestry that forms the basis of a high level name, and considerable judgment must be exercised, in addition to phylogenetic scrutiny, to decide how much this hypothesis can be modified before the name is sufficiently compromised to demand synonymy.

For the high-level clades within Mammalia, the best set of guiding principles for high-level taxonomy are the introductory pages of Simpson [8], reflected also in McKenna and Bell [9]. Since the early 1990s, several investigators have to varying degrees proposed a departure from the ICZN and its Linnean basis in the form of the "Phylocode" [10], which would entail a system of official arbitration for high-level categories. Indeed, one of the principles of the Phylocode is the formal recognition that Linnean ranks are arbitrary. However, one need not abandon the Linnean system, or even depart substantially from historical practice, to incorporate this recognition into nomenclature [11-15]. Like the ICZN, Phylocode also emphasizes priority in recognizing taxon names (cf. article 12 of [10]). Following Simpson (pp. 27-28 in [8]), "article 25 of the [ICZN, now article 23 in the 1999 edition] is the famous Law of Priority, which is the basic principle and the storm center of technical nomenclature.... While fully agreeing that the [ICZN] badly needs revision ... I have attempted to follow their letter exactly in [my 1945] classification of mammals. Where the letter is ambiguous, I have taken the spirit to be that choice should promote stability and perpetuate common usage as far as possible." Hence, Simpson viewed "stability and common usage" as the other major criteria to adjudicate among competing names. While Simpson regarded priority as a fundamental principle, stability is also of major importance, as demonstrated by the fact that the ICZN regularly issues rulings overturning priority to maintain stability (published in the *Bulletin of Zoological Nomenclature*).

**Figure 1:**
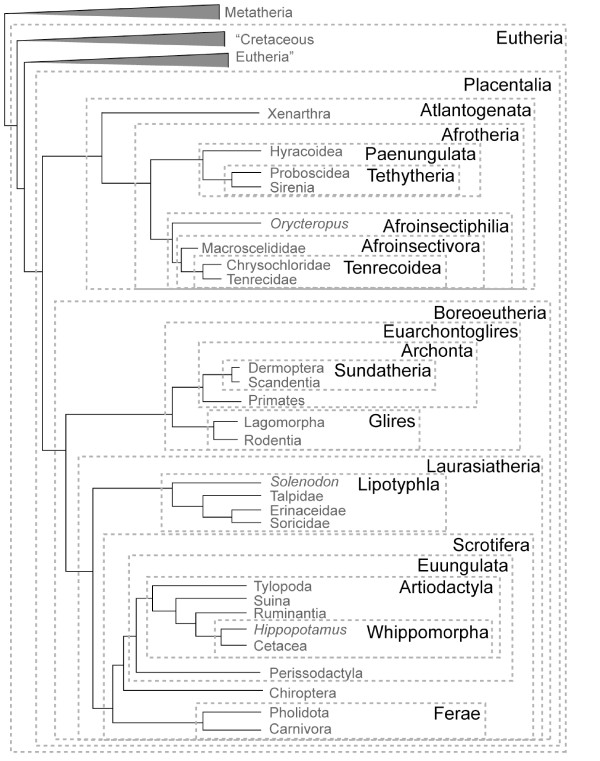
**Summary of placental mammal taxonomy based on the phylogeny of **[2,3]. Authorship and content for high-level clades is given in Table 1; names for some competing hypotheses not shown here [e.g.,[29,30,78]] are given in Table 2.

A plethora of new names have been applied to high-level mammalian clades in recent years (summarized in Fig. 1 and Tables 1 and 2). In some cases, these names have not been applied with regard to the taxonomic conventions outlined by Simpson [8]. Dispute about nomenclature is unfortunate, but given the context of an increasingly stable phylogenetic tree for living Mammalia, there are worse problems in biology than relatively minor disagreements about what to call the large number of now widely-recognized, high-level placental mammalian clades. Nevertheless, some of this new nomenclature can be very confusing. Arbitration among these names is ongoing and, it seems, popularity will play a larger role than reliance on Simpson [8] or similar efforts based on principle.

**Table 1:** Summary of mammalian taxonomic terms based on Fig. 1 [2,4], updating references given in [5] with corrections denoted by asterisks.

Taxon with priority	Content	Synonyms
Placentalia[70]	All descendants of last common ancestor of sloth, tenrec, human, horse	

Atlantogenata[17]	Xenarthra, Afrotheria	Xenafrotheria[49] Notoplacentalia[16]

Afrotheria[19]	Paenungulata, Afroinsectiphilia	Afroplacentalia[16]

Afroinsectiphilia[31]	Tubulidentata, Afroinsectivora	Fossoromorpha [[71]*]

Afroinsectivora[31]	Tenrecoidea, Macroscelididae	Haemochorialia[33]

Tenrecoidea[27]	Tenrecidae, Chrysochloridae	Afrosoricida[19] Tenrecomorpha[78]

Paenungulata[8]	Hyracoidea, Tethytheria	Uranotheria[9]

Tethytheria[22]	Proboscidea, Sirenia	

Boreoeutheria [[72]*]	Laurasiatheria, Euarchontoglires	Boreotheria[31] Boreoplacentalia[16]

Laurasiatheria[17]	Lipotyphla, Scrotifera	Laurasiaplacentalia[16]

Scrotifera[17]	Ferae, Chiroptera, Euungulata	Variamana [[71]*]

Euungulata[31]	Artiodactyla, Perissodactyla	

Artiodactyla [[73]*]	Whippomorpha, Ruminantia, Tylopoda, Suiformes	Cetartiodactyla[35] Eparctocyona[9]

Ferae [9]	Carnivora, Pholidota	Ostentoria[74]

Whippomorpha[17]	Cetacea, Hippopotamidae	Cetancodonta[78]

Lipotyphla[75]	Erinaceidae, Talpidae, Soricidae, Solenodontidae	Eulipotyphla[17]

Euarchontoglires[1]	Archonta, Glires	Archontoglires[16] Supraprimates[31]

Glires[76]	Lagomorpha, Rodentia	

Archonta[21]	Primates, Scandentia, Dermoptera	Euarchonta[17]

Sundatheria[32]	Scandentia, Dermoptera	Paraprimates[33]

Author attributions are based on the first use of the clade with its current, or near-current, meaning. For example, Butler [77] used "Tenrecomorpha" but did not include chrysochlorids. In contrast, Arnason et al. [78] used this term to indicate the tenrec-golden mole clade as it is currently-understood and are therefore cited as the authors for that term as a junior synonym of the name with precedence, Tenrecoidea McDowell 1958 [27]. Some additional clades apparent in recent studies [e.g., [29,30]], but not reflected in the summary topology of Fig. 1, are listed in Table 2. Note that several names identified as crown-level synonyms (e.g., Uranotheria, Eparctocyona) remain potentially useful as stem counterparts to the accepted crown taxa.

**Table 2:** Taxonomic designations for named clades with potential support [e.g., [29,30,78]] not depicted in Fig. 1.

Taxon with priority	Content	synonym
Exafroplacentalia [31]	All placental mammals except Afrotheria	Notolegia [71]

Epitheria [22]	All placental mammals except Xenarthra	

Pseudoungulata [17]	Tubulidentata-Paenungulata	

Primatomorpha [34]	Dermoptera-Primates	

Dermosimii [78]	Dermoptera-Anthropoidea	

Pegasoferae [29]	Perissodactyla-Ferae-Chiroptera	

Fereuungulata [17]	Artiodactyla-Perissodactyla-Ferae	

Zooamata [17]	Perissodactyla-Ferae	

Cetferungulata [35]	Carnivora, Perissodactyla, Artiodactyla	

Here, we suggest how an application of Simpson's guidelines can help discriminate among competing names for high-level groups of placental mammals. In a nutshell, priority and stability should comprise the overriding principles by which new, high-level taxa are named. Established names for any given clade should not be altered unless the name with precedent unambiguously threatens stability. We suggest that the most appropriate will be those that are 1) the first, published name for a monophyletic group with unique content and 2) based on terms deemed familiar and logical to as many students as possible. Failure of a given taxon to meet one of these criteria does not necessarily mean it is invalid, but failure in both should.

### Names for a resolved tree

Arnason et al. [16] suggested several names for mammalian taxa using etymological and orthographic criteria. For example, in their view the unusual term Whippomorpha Waddell et al. 1999 [17] for the hippo-whale clade should be replaced by Cetancodonta Arnason et al. 2000 [18], based on potential confusion with hippomorph perissodactyls. In addition, they argued that Laurasiatheria Waddell et al. 1999 [17] and Afrotheria Stanhope et al. 1998 [19] should be replaced by Laurasiaplacentalia Arnason et al. 2008 [16] and Afroplacentalia Arnason et al. 2008 [16], as the "placentalia" endings more accurately reflect the status of these clades as crown placental mammals [20]. Arnason et al. [16] are admirably reluctant to accept prefixes of high-level taxa that have only been partly modified by recent systematic work. For example, as originally defined [21], Archonta differs from its modern incarnation in the position of chiropterans and macroscelidids. As used by McKenna [22], Archonta excluded macroscelidids and was even closer to the modern version, which groups primates, dermopterans, and scandentians together. Other researchers [1] have dubbed this modified version of Gregory's clade Euarchonta, and correspondingly use Euarchontoglires for the next more basal node that joins archontans with lagomorphs and rodents. A legitimate interpretation of Simpson [8] (see in particular his point #30, p. 33) would be consistent with the avoidance of such prefixes in cases where the content of a clade has not drastically changed. Some may argue that the current modifications to Gregory's Archonta are in fact drastic, but the no-less-drastic recognition that birds fall within Mesozoic Dinosauria has not led to the novel taxa "Eudinosauria" or "Avesdinosauria" [23], nor has the incorporation of pinnipeds into Carnivora resulted in a change to the latter term [24]. Hence, this would support retaining Archonta over "Euarchonta", Lipotyphla over "Eulipotyphla", and Artiodactyla over "Cetartiodactyla".

### Balancing priority and stability

While Arnason et al. [16] have identified etymological and grammatical points in support of their mammalian taxonomy, they are frequently inconsistent with Simpson's [8] emphasis on priority. As summarized above, there is substantial precedent for favoring priority over other concerns. For example, Florentino Ameghino [25,26] was convinced that his native Argentina was the origin of certain clades of mammals, such as equids and primates. As South America's most prolific paleontologist, this conviction expressed itself in many of the names he published. We continue to accept and use names such as *Notohippus *Ameghino 1901 [25] and *Archaeopithecus *Ameghino 1897 [26] for endemic South American ungulates, even though they have relatively little to do with equids and anthropoid primates (respectively), because of the importance of article 23 of the ICZN, which values priority over other factors.

In the same way that we are compelled to accept Ameghino's references to primates and equids among names for unrelated, endemic South American mammals, supraordinal taxa similarly deserve recognition based in the first instance on priority, even if this involves questionable orthography and/or etymology. Hence, genuinely novel clades should be known by their earliest published names. For mammals, this includes Laurasiatheria, Boreoeutheria, Afrotheria, Atlantogenata, Scrotifera, and Euarchontoglires (Fig. 1), assuming the phylogenetic basis for these taxa remains stable (cf. Table 2). Even though some may find orthographic and/or etymological reasons to regret the choice of one or more of these names, alternatives (e.g., Notoplacentalia Arnason et al. 2008 [16] or Xenafrotheria Asher 2005 [49]) should be discarded, or at best retained as stem designations, in favor of their senior synonyms (e.g., Atlantogenata Waddell et al. 1999 [17]).

Several mammalian clades have a long history among taxonomists, and have been recognized as such in most recent studies. These include Paenungulata, Glires, Archonta, and most individual orders within Mammalia [23]. More problematic are names that have been overlooked in recent publications, such as Eparctocyona McKenna 1975 [22], Uranotheria McKenna and Bell 1997 [9], Tenrecoidea McDowell 1958 [27], and Zalambdodonta Gill 1883 [28]. The remainder of this paper summarizes the case for placental mammal nomenclature based on the phylogenetic tree depicted in Fig. 1 (see also Table 1), derived from [2-4]. While this tree is relatively stable [5], we acknowledge that debate continues at certain nodes [29,30], and list some alternative terms in Table 2. Such potential for topological change underscores the need to publish synonymies that detail previous meanings of high-level names (Tables 1 and 2), essential to understand their usage through time and to clarify their past and current applications.

#### Euarchontoglires

As discussed above, Archonta is preferable to "Euarchonta" [23]. However, dropping the "eu" in that term would not affect the name Euarchontoglires, which was erected for a genuinely novel concept and has no precedent in the systematic literature. Waddell et al. [31] proposed the name Supraprimates for this assemblage in an article published on 17 December 2001 (as confirmed by the *Genome Informatics *editorial office), a few days after Euarchontoglires was published on 14 December 2001 by Murphy et al. [1]. The latter name therefore has precedence, at least for its intended crown constituents. Sundatheria Olson et al. 2005 [32] as a designation for Scandentia-Dermoptera similarly has precedence over Paraprimates Springer et al. 2007 [33]. Both may be rendered superfluous if the competing hypothesis of Dermoptera-Primates (Primatomorpha Beard 1993 [34]), supported by analyses of genomic indels [30], proves robust in future analyses.

#### Laurasiatheria

Archibald [23] made the point that Ferungulata of Simpson [8], including terrestrial artiodactyls, perissodactyls, carnivorans, paenungulates, as well as extinct "condylarths" and South American ungulates (but not pholidotans or cetaceans), would be preferable to Fereuungulata Waddell et al. 1999 [17]. However, because the concepts of Simpson vs. Waddell differ substantially in content, particularly regarding paenungulates and cetaceans, this is arguably a case where interpretation of Simpson's points 24, 29, and 30 ([8], pp. 32-33) justify a change in taxon name, should the underlying phylogeny of Waddell et al. [17] prove correct. "Ferungulata" was also used by Montgelard et al. [35], but like Simpson they also included sirenians and proboscideans in this clade, and excluded the counterintuitive but well-supported position of chiropterans among laurasiatheres near northern, ungulate-grade mammals [3].

Arguably, therefore, Waddell's concept would be preferable here too. Favoring Euungulata Waddell et al. 1999 [17] over Ungulata McKenna 1975 [22] is based on the similar fact that the latter concept differs from current phylogenies by including both tubulidentates and paenungulates among "ungulates". Hence, Ferungulata Simpson 1945 [8], Ungulata McKenna 1975 [22], and Ferungulata Montgelard et al. 1997 [35] represent substantially different concepts of common descent than those currently supported by recent mammalian phylogenies (e.g., Fig. 1), and therefore may be replaced with new names [17] based on Simpson's criteria outlined above.

#### Cetaceans and artiodactyls

The first published name for the hippo-whale clade was Whippomorpha Waddell et al. 1999 [17]. By the early 1990s, the intra-artiodactyl affinity of Cetacea had been suggested [36], with some publications correctly identifying Hippopotamidae as the cetacean sister group [37,38]. Montgelard et al. [35] figured the hippo-whale branch with the label "Cetacea + Ancodonta", but in our view this falls short of creating an explicit nomen for hippo-whale. Arnason et al. [18] pointed out the potential confusion of "Whippo-" with Hippomorpha, a clade consisting of equid perissodactyls and their fossil relatives. While this is a legitimate concern, by itself it is insufficient to overturn the clear priority of Waddell's term. This case is a high-level, taxonomic analogy with the retention of Ameghino's confusing names for genera of South American notoungulates. Hence, a fair application of Simpson [8] to this issue, with his emphasis on priority, means that Whippomorpha Waddell et al. 1999 [17] should be the accepted name for the whale-hippo crown clade.

The name for the larger assemblage of terrestrial artiodactyls plus Cetacea nested within it is slightly more problematic. As previously stated, stability would be served by retaining the name Artiodactyla over its frequently used alternative, "Cetartiodactyla", despite the addition of cetaceans. While addition of the "cet" prefix has become very popular in the mammalian systematics literature, Artiodactyla as a published ordinal designation is still widespread, with over 15000 hits on google scholar from 2000-2009, vs. 482 for "Cetartiodactyla". The advantages of formalizing the new and very well supported systematic position of whales in its high-level taxonomic designation are considerable. Furthermore, given the fact that in some morphological aspects (dentition, body size) whales are more varied than even-toed ungulates classically arranged within Artiodactyla, a name change may be warranted under Simpson's criterion for "reasonable emendation" ([8] p. 33, point 30A). However, such considerations do not change the fact that including whales within Artiodactyla is analogous to cases mentioned above (e.g., Dinosauria including birds) in which the content of the clade in question has not drastically changed and high-level names have not been altered. Therefore, we suggest it would be most consistent to retain Artiodactyla (including cetaceans) as a taxon rather than changing the ordinal name for this group, the even-toed, terrestrial constituents of which remain intact [23,24].

Eparctocyona was used by McKenna [22] and McKenna and Bell [9] to denote not only cetaceans and artiodactyls, but also assemblages of so-called "condylarths", including mesonychids, arctocyonids, and other extinct groups with controversial affinities to living orders. Based on priority, the name Eparctocyona would trump other, recent candidates for the whale-even-toed ungulate assemblage, such as Cetartiodactyla Montgelard et al. 1997 [35], and has been used in a few publications [9,22,39,40]. Indeed, as a stem clade designation, including for example one or more extinct "condylarths" as sister taxa to Artiodactyla, it may yet prove appropriate. However, as a crown designation it is still a junior synonym of Artiodactyla, and it entails the controversial implication that multiple, poorly understood fossil assemblages ("condylarths" such as hyopsodontids, phenacodontids, arctocyonids, et al.) comprise artiodactyl relatives to the exclusion of other mammals.

#### Paenungulata

Uranotheria [9] differs from Paenungulata of Simpson [8] in that it excludes several extinct groups, such as pyrotheres, pantodonts, and dinoceratans. However, in terms of its living constituents, the two clades are the same: proboscideans, sirenians, and hyracoids. Taxonomic stability is positively served by not changing a taxon name with every alteration of its contents (cf. Archonta excluding bats, Dinosauria including birds). Hence, the exclusion of some fossil clades from Simpson's Paenungulata does not justify the wholesale replacement of that nomen, particularly given its priority. Although a few authors have followed McKenna and Bell [9] in using Uranotheria [41], and this designation may still be useful to denote a stem clade, retaining Paenungulata to signify Proboscidea-Sirenia-Hyracoidea better serves the spirit of taxonomic practice outlined by Simpson [8]).

#### Tenrecids and chrysochlorids

Both Zalambdodonta Gill 1883 [28] and Tenrecoidea McDowell 1958 [27] have been used to signify a tenrecid-chrysochlorid clade, although Gill included the Caribbean *Solenodon *in his formulation. Zalambdodont also has an anatomical meaning, indicating a single, upper "V"-shaped molar loph that occludes with lower molars that lack a complete talonid basin [42]. In addition to tenrecids, chrysochlorids, and *Solenodon*, dental zalambdodonts also include some metatherians and extinct groups such as apternodontids [43]. As used by McDowell [27] and Frost et al. [44], Tenrecoidea includes only tenrecids and chrysochlorids; but the root has frequently appeared over the last century with various suffixes and biological meanings appended to it [45], a fact which has led some investigators [46-48] to use the term "Tenrecomorpha" instead. The seminal paper of Stanhope et al. [19], in which Afrotheria was first named, included the taxon "Afrosoricida" for the tenrec-chrysochlorid clade with no justification for why their novel term should replace one that had the same content, or why their major discovery that African insectivorans comprised an entirely different mammalian radiation, apart from holarctic insectivorans such as soricids, should nevertheless be given almost the same name. Possibly Stanhope et al. [19] were simply unaware of McDowell's Tenrecoidea, not unlike the first author's (RJA) neglect [49] of Atlantogenata Waddell et al. 1999 [17] for the Xenarthra-Afrotheria clade, which has priority over Xenafrotheria Asher 2005 [49] as a crown designation.

In any event, "Afrosoricida" is a name with misleading taxonomic implications, implying a family group association with *Afrosorex*, a subgenus (now synonymized) of *Crocidura *[50,51]. Furthermore, a number of biologists have already recognized Tenrecoidea sensu McDowell [23,44,52-55]. Based on priority alone, Zalambdodonta Gill 1883 [28] might also be regarded as a contender; however, this term is even less familiar to contemporary zoologists and has never been used for just tenrec-golden mole. Arguments in favor of "Afrosoricida" have been made by authors [56-58] who regard the historical baggage and implied Linnean rank of Tenrecoidea as sufficient to reject it in favor of the more recent term "Afrosoricida". Indeed, as of this writing "Afrosoricida" generates more hits on google-scholar (214) than either Tenrecoidea (141) or Zalambdodonta (34). Further in its favor is the fact that "Afrosoricida" does not imply close relations with non-afrotherian taxa such as apternodontids and *Solenodon*. As mentioned above, it replaces these with a confusing allusion to modern soricids. For new students, the potential confusion of "tenrecoid" with *Solenodon *and apternodontids would seem substantially more remote than that of "afrosoricid" with soricids, such as *Afrosorex*.

To make matters more complicated, a few authors [59,60] use "Tenrecoidea" with the same meaning as the Tenrecidae of most other mammalogists [58]. That is, they elevate Malagasy tenrecs to "Tenrecidae" and African potamogalines to "Potamogalidae" and refer to both as "Tenrecoidea". This practice has precedent because older uses of the family-level name Tenrecidae did not include potamogalines [8,61], and it is a positive step insofar as it recognizes the monophyly of the extant Malagasy forms to the exclusion of mainland potamogalines [62-64]. However, elevation of tenrecs and golden moles to Linnean ranks above the family is based on the now obsolete notion that the two groups are unrelated [65]. Furthermore, the recognition of Malagasy tenrec monophyly could just as easily have been done by denoting taxa at levels below the Linnean family, preserving both the now-common understanding of the term Tenrecidae [9,51,62-64,66] and the precedent of McDowell's [27] Tenrecoidea as a designation for tenrecid-chrysochlorid.

**Figure 2:**
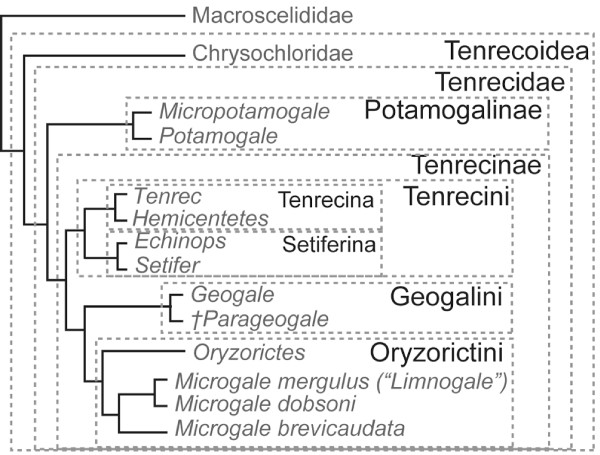
**Proposed taxonomy for afroinsectivoran mammals, maintaining common understanding of Tenrecidae **[66]** and priority of Tenrecoidea McDowell 1958 **[27]**, based on the phylogeny of **[62-64].

Such a proposal for nomenclature within the extant Afroinsectivora is shown in Fig. 2. Here, Malagasy tenrecs are formally recognized in the Tenrecinae and mainland African tenrecs in the Potamogalinae. Spiny tenrecs are grouped in the Tenrecini, *Geogale *in the Geogalini, and other soft tenrecs in the Oryzorictini, with *Limnogale *recognized as a synonym of *Microgale *[62]. To date, the largest analysis of sequence data for living tenrecs [64] supports the placement of *Geogale *as sister taxon to *Microgale *(including *Limnogale*) and *Oryzorictes*. The possibility that one or more African fossils are more closely related to *Geogale *than to other tenrecs [63] justifies placement of the Miocene, African *Parageogale *with *Geogale *in the Geogalini. Further suprageneric groupings could be similarly be made within tenrecins and oryzorictins (e.g., Tenrecina for *Tenrec *and *Hemicentetes*). This arrangement better recognizes common use (cf. Tenrecidae of [66]) and priority (cf. Tenrecoidea McDowell 1958 [27]) than the alternative taxonomy [59,60] in which higher, rather than lower, ranks are used to recognize Malagasy tenrec monophyly. Furthermore, such a taxonomy for tenrecs is consistent with the analogous case of the tribe Hominini now used for *Australopithecus, Paranthropus *and other habitually bipedal primates, formerly referred to as "hominids", which are more closely related to *Homo *than to *Pan *or *Gorilla *[67].

#### Linnean rank and priority

Linnean ranks were important to Simpson [8], although he recognized that they are without intrinsic biological meaning. Simpson regarded it convenient to reserve some taxonomic endings for certain levels of the hierarchy not for any intrinsic meaning of ranks, but only because this practice maximizes stability, particularly the suffix "idae" at the familial rank. Importantly, although Bronner and Jenkins [58] regard the "oidea" of Tenrecoidea as a major criterion for rejecting its use to designate the (arbitrary) ordinal status of the chrysochlorid-tenrecid clade, Simpson did not regard "oidea" as exclusively a superfamilial ending and used it at least once as an ordinal suffix (Hyracoidea). Nor would Simpson have regarded early use of Tenrecidae (including his own [45]) as prohibiting subsequent alterations of rank or content, reflecting article 23.3.1 of the ICZN [6], which notes that "priority of the name of a nominal taxon is not affected by elevation or reduction in rank". Simpson ([8], p. 32) agreed with retaining taxon authorship for a name used at a different level of the Linnean hierarchy, and recognized that rank-reshuffling is not necessarily an act of creativity deserving of a reattributed citation. However, when "a basic change in group concept is also made", i.e., the content of a given taxon changes substantially, he condones reattributed authorship ([8], p. 32). It is therefore reasonable to interpret Simpson [8] in favor of the view that rank and homogeneity of taxon suffixes are less important than priority and stability for arbitrating among names.

For these reasons, i.e., the same ones that compel use of Ameghino's generic or Waddell's supraordinal names (due to priority), Gregory's Archonta or Owen's Dinosauria (despite changes within each taxon), we regard Afrosoricida Stanhope et al. 1998 [19] as a junior synonym of Tenrecoidea McDowell 1958 [27].

## Conclusions

Systematists now have an unprecedented understanding of how clades of mammals are interrelated. Arguments about nomenclature are a side-effect of this positive state of affairs, a welcome change from the days, not so long ago, when decades of study did not lead to broad consensus on the affinities of certain high-level clades [68,69]. We hope the cases discussed above will help to illuminate the standards by which systematists should provide names to novel high-level clades. Not all readers will agree with our recommendations, in which decisions have been made about the degree to which a an existing phylogenetic concept must change in order to justify a corresponding change in name. In some instances (e.g., replacing Ferungulata Simpson 1945 with Fereuungulata Waddell et al. 1999), we argue that a new name is justified; in others (e.g., replacing Tenrecoidea McDowell 1958 with Afrosoricida Stanhope et al. 1998) we argue that it is not. Regardless of disagreements over individual cases, we hope that our larger point is broadly accepted, i.e., that new names should be coined with great reluctance, relying whenever possible on existing terms, following Simpson's emphases on priority and stability. Our expectation is that an ever improving understanding of the molecular, phenotypic, and paleontological diversity of mammals will result in the discovery of yet more such clades, which will require more names. Our hope is that the nomenclature applied to this increased diversity will be principled, rather than populist.

## Authors' contributions

RA conceived of the study and wrote the first draft; KH made substantial corrections and additions to the text. Both authors read and approved the manuscript.
